# The Source and Pathophysiologic Significance of Excreted Cadmium

**DOI:** 10.3390/toxics7040055

**Published:** 2019-10-18

**Authors:** Soisungwan Satarug, David A. Vesey, Werawan Ruangyuttikarn, Muneko Nishijo, Glenda C. Gobe, Kenneth R. Phelps

**Affiliations:** 1 Kidney Disease Research Collaborative, The University of Queensland Faculty of Medicine and Translational Research Institute, Woolloongabba, Brisbane 4102, Australia; David.Vesey@health.qld.gov.au (D.A.V.); g.gobe@uq.edu.au (G.C.G.); 2 Department of Nephrology, Princess Alexandra Hospital, Brisbane 4075, Australia; 3 Division of Toxicology, Department of Forensic Medicine, Chiang Mai University, Chiang Mai 50200, Thailand; ruangyuttikarn@gmail.com; 4 Department of Public Health, Kanazawa Medical University, Uchinada, Ishikawa 920-0293, Japan; ni-koei@kanazawa-med.ac.jp; 5 School of Biomedical Sciences, The University of Queensland, Brisbane 4072, Australia; 6 NHMRC Centre of Research Excellence for CKD.QLD, UQ Health Sciences, Royal Brisbane and Women’s Hospital, Brisbane 4029, Australia; 7 Stratton Veterans’ Affairs Medical Center and Albany Medical College, Albany, NY 12208, USA; Kenneth.Phelps@va.gov

**Keywords:** β_2_-microglobulin, cadmium, creatinine clearance, glomerular filtration, N-acetyl-β-d-glucosaminidase, nephron mass, nephrotoxicity

## Abstract

In theory, the identification of the source of excreted cadmium (Cd) might elucidate the pathogenesis of Cd-induced chronic kidney disease (CKD). With that possibility in mind, we studied Thai subjects with low, moderate, and high Cd exposure. We measured urine concentrations of Cd, ([Cd]_u_); N-acetyl-β-d-glucosaminidase, a marker of cellular damage ([NAG]_u_); and β_2_-microglobulin, an indicator of reabsorptive dysfunction ([β_2_MG]_u_). To relate excretion rates of these substances to existing nephron mass, we normalized the rates to creatinine clearance, an approximation of the glomerular filtration rate (GFR) (E_Cd_/C_cr_, E_NAG_/C_cr_, and E_β2MG_/C_cr_). To link the loss of intact nephrons to Cd-induced tubular injury, we examined linear and quadratic regressions of estimated GFR (eGFR) on E_Cd_/C_cr_, eGFR on E_NAG_/C_cr_, and E_NAG_/C_cr_ on E_Cd_/C_cr_. Estimated GFR varied inversely with both ratios, and E_NAG_/C_cr_ varied directly with E_Cd_/C_cr_. Linear and quadratic regressions of E_β2MG_/C_cr_ on E_Cd_/C_cr_ and E_NAG_/C_cr_ were significant in moderate and high Cd-exposure groups. The association of E_NAG_/C_cr_ with E_Cd_/C_cr_ implies that both ratios depicted cellular damage per surviving nephron. Consequently, we infer that excreted Cd emanated from injured tubular cells, and we attribute the reduction of eGFR to the injury. We suggest that E_Cd_/C_cr_, E_NAG_/C_cr_, and eGFR were associated with one another because each parameter was determined by the tubular burden of Cd.

## 1. Introduction

Cadmium (Cd), a divalent metal used for industrial purposes, is an important environmental pollutant in some regions of the world [1,2,3,4,5]. The metal is conveyed to humans in food, air, and tobacco smoke, and subsequently gains access to the circulation through the gut and lungs [5]. Salts of ionized Cd are absorbed in the duodenum; in addition, complexes of Cd with plant metallothioneins (MT) and phytochelatins (PC) may be absorbed in the colon after liberation by bacteria [6]. In the bloodstream, Cd is bound to red blood cells, albumin, glutathione (GSH), sulfur-containing amino acids, MT, and PC [6,7,8,9,10]. In the liver, hepatocytes take up Cd not bound to MT [10], synthesize MT in response to the metal, and store complexes of CdMT. These complexes are subsequently released from hepatocytes and transported to the kidneys [8,11,12]. Cd in plasma is filterable by glomeruli if it is bound to GSH, amino acids, MT, or PC [8,9], but the fraction of circulating Cd that enters the filtrate is unknown. The proximal tubule reabsorbs and retains most or all of the filtered Cd with an array of channels, solute carriers, and mediators of endocytosis [7,10,13,14,15,16]. Basolateral uptake may also add to the cellular content of Cd in the proximal tubule [16,17,18].

It is currently assumed that the magnitude of a gradually acquired burden determines the toxicity of Cd in tubular cells [19]. The emergence of Cd from lysosomes induces robust intracellular synthesis of MT, which greatly mitigates the injury inflicted by free Cd through complexation of the metal [20]. Nevertheless, a fraction of Cd remains unbound to MT and is presumed to promote autophagy, apoptosis, and necrosis as accumulation of Cd progresses [19,21]. Manifestations of renal toxicity include increased excretion of cellular proteins, impaired reabsorption of filtered substances, histologically demonstrable tissue injury, loss of intact nephrons, and reduction of the glomerular filtration rate (GFR) [5,8,21,22,23,24,25,26,27]. GFR may continue to fall for many years after exogenous exposure ceases [22,24], presumably because traffic of CdMT from the liver to kidneys persists.

In human studies of Cd-induced nephropathy, the most commonly assayed marker of tubular cell damage is the lysosomal enzyme N-acetyl-β-d-glucosaminidase (NAG). Because NAG is too large to be filtered by glomeruli, excessive excretion (E_NAG_) signifies tubular injury [28]. The most commonly measured indicator of impaired reabsorption is β_2_-microglobulin (β_2_MG). This small circulating protein is extremely filterable by glomeruli [29]; ordinarily, more than 99% of filtered β_2_MG is reabsorbed [30], but that percentage falls early in the course of tubular injury [31].

Although the urinary excretion rate of Cd (E_Cd_) is believed to reflect the body burden of the metal [5], the precise source and pathophysiologic significance of excreted Cd have not been clarified. One possibility is that Cd is excreted because it is filtered and not reabsorbed; an alternate possibility is that excretion reflects liberation of Cd into filtrate from injured or dying tubular cells [32]. This distinction is important because the source of urinary Cd is central to the relationship between Cd accumulation and progression of chronic kidney disease (CKD). Herein, we present evidence that excreted Cd emanates from cells that it has injured. The injury leads to the loss of intact nephrons, reduction of GFR, and impaired reabsorption of filtered β_2_MG.

## 2. Materials and Methods

### 2.1. Study Subjects

The Institutional Ethical Committees of Chulalongkorn University, Chiang Mai University and the Mae Sot Hospital approved the study protocol (Approval No. 142/2544, 5 October 2001) [33]. All participants gave informed consent before participation. Subjects were recruited from urban communities in Bangkok in 2001/2002 and from subsistence farming areas in Mae Sot District, Tak Province, Thailand in 2004/2005 [33]. They had lived at their current addresses for at least 30 years. Exclusion criteria were pregnancy, breast-feeding, a history of metal work, and a hospital record or physician’s diagnosis of an advanced chronic disease. Because occupational exposure was an exclusion criterion, we presumed that all participants had acquired Cd from the environment.

Cd exposure was low in Bangkok and moderate or high in Mae Sot [33,34]. Determination of exposure was based on reported levels of Cd in rice grains grown in the Cd-affected areas of the Mae Sot District [35,36]. After exclusion of subjects with incomplete datasets, we studied 172, 310, and 222 persons from the low, moderate, and high exposure areas, respectively. 

### 2.2. Collection of Biological Specimens and Laboratory Analyses 

Second morning-void urine samples were collected after an overnight fast. Within three hours after urine sampling, specimens of whole blood were obtained and serum samples were prepared. Aliquots of urine, whole blood, and serum were transported on ice from a mobile clinic to a laboratory and stored at −20 °C or −80 °C for later analysis. The assay for urine and serum creatinine concentrations ([cr]_u_, [cr]_p_]) was based on the Jaffe reaction. The urine NAG assay was based on colorimetry (NAG test kit, Shionogi Pharmaceuticals, Sapporo, Japan). The urine β_2_MG assay was based on the latex immunoagglutination method (LX test, Eiken 2MGII; Eiken and Shionogi Co., Tokyo, Japan). When the urine concentration of β_2_MG ([β_2_MG]_u_) was below the limit of detection (LOD), 0.5 µg/L, the value assigned to [β_2_MG]_u_ was LOD/(square root of 2).

For the Bangkok group, [Cd]_u_ was determined by inductively-coupled plasma mass spectrometry (ICP/MS, Agilent 7500, Agilent Technologies), because it had the high sensitivity required to measure Cd concentrations below the detectable limit of atomic absorption spectrophotometry. Multi-element standards (EM Science, EM Industries, Inc., Newark, NJ, USA) were used to calibrate Cd analyses. The accuracy and precision of those analyses were evaluated with reference urine (Lyphochek^®^, Bio-Rad, Sydney, Australia). When [Cd]_u_ was less than the detection limit of 0.05 μg/L, the concentration assigned was the detection limit divided by the square root of 2.

For the Mae Sot groups, [Cd]_u_ was determined by atomic absorption spectrophotometry (Shimadzu Model AA-6300, Kyoto, Japan). Urine standard reference material No. 2670 (National Institute of Standards, Washington, DC, USA) was used for quality assurance and control purposes. None of the urine samples from the Mae Sot groups were found to have [Cd]_u_ below the detection limit.

### 2.3. Estimated Glomerular Filtration Rate (eGFR)

The glomerular filtration rate was estimated with equations from the Chronic Kidney Disease Epidemiology Collaboration (CKD-EPI) [37,38]. CKD stages 1, 2, 3, 4, and 5 corresponded to eGFR of 90–119, 60–89, 30–59, 15–29, and <15 mL/min/1.73 m^2^, respectively. For dichotomous comparisons, CKD was defined as eGFR < 60 mL/min/1.73 m^2^.

### 2.4. Normalization of Excretion Rates to Creatinine Clearance (C_cr_)

Excretion rates of Cd, NAG, and β_2_MG were normalized to C_cr_ to yield the ratios E_Cd_/C_cr_, E_NAG_/C_cr_, and E_β2MG_/C_cr_ in units of mass (amount excreted) per volume of filtrate. For *x* = Cd, NAG, or β_2_MG, E*_x_*/C_cr_ was calculated as [*x*]_u_[cr]_p_/[cr]_u_ ([39]; Supplementary Materials).

### 2.5. Statistical Analysis

Data were analyzed with SPSS 17.0 (SPSS Inc., Chicago, IL, USA). Distributions of excretion rates for Cd, NAG, and β_2_MG were examined for skewness, and those showing rightward skewing were subjected to base-10 logarithmic transformation before analysis. Departure of a given variable from normal distribution was assessed with the one-sample Kolmogorov–Smirnov test. For continuous variables not conforming to a normal distribution, the Kruskal–Wallis test was used to determine differences among the three localities in E_Cd_/C_cr_, E_NAG_/C_cr_, E_β2MG_/C_cr_, and other parameters. The Mann–Whitney *U*-test was used to compare mean differences between two groups. The Chi-Square test was used to determine differences in percentage and prevalence data. *p*-values ≤ 0.05 for two-tailed tests were assumed to indicate statistical significance.

Polynomial regression was used to fit lines and curves to the scatterplots of five pairs of variables, including eGFR versus E_Cd_/C_cr_, eGFR versus E_NAG_/C_cr_, E_NAG_/C_cr_ versus E_Cd_/C_cr_, E_β2MG_/C_cr_ versus E_Cd_/C_cr_, and E_NAG_/C_cr_ versus E_β2MG_/C_cr_. A linear model, *y = a + bx*, was adopted if the relationship was monotonic. A quadratic model (second-order polynomial), *y* = *a* + *b*_1_*x* + *b*_2_*x*^2^, was used if there was a significant change in the direction of the slope (*b*_1_ to *b*_2_) for prediction of the dependent variable *y*. In both types of equations, *a* represented the *y*-intercept.

The relationships between *x* and *y* were assessed with *R*^2^ (the coefficient of determination) and with unstandardized and standardized β coefficients. In linear and quadratic models, *R*^2^ is the fraction of variation in *y* that is explained by the variation in *x*. In linear models, the unstandardized β coefficient is the slope of the linear regression, and the standardized β coefficient indicates the strength of the association between *y* and *x* on a uniform scale. To examine quadratic curves relating eGFR to E_Cd_/C_cr_ and E_NAG_/C_cr_, we performed slope change analyses with a linear regression method.

## 3. Results

Table 1 presents data concerning age, gender, blood pressure, smoking status, and renal function of subjects with low, moderate, and high environmental exposure to Cd. There were significant differences in age and percentages of women and smokers across the three exposure subsets. Female gender was overrepresented in the moderate exposure group. More than half of subjects in the high exposure group were smokers. Blood pressures were recorded in the low and moderate exposure groups; systolic and mean pressures were significantly higher in the latter.

Mean eGFR fell and the percentages of stages 2 and 3 CKD rose with intensity of exposure. The mean serum creatinine concentration was higher in the high-Cd locality. In general, urine concentrations of Cd, NAG, and β_2_MG rose with Cd exposure, but [NAG]_u_ and [β_2_MG]_u_ were higher in the moderate Cd group than in the other two. E_Cd_/C_cr_, E_NAG_/C_cr_, and E_β2MG_/C_cr_ followed the same pattern.

**Figure 1:**
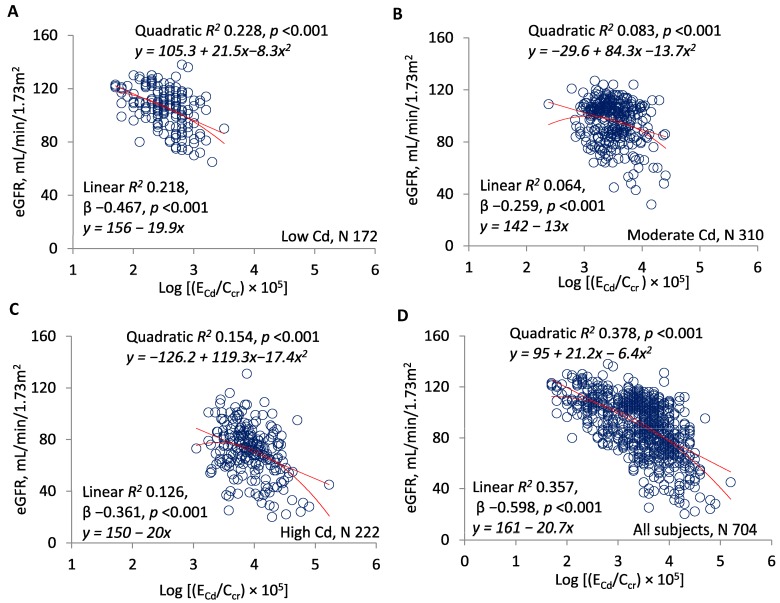
E_Cd_/C_cr_ as a predictor of the estimated glomerular filtration rate (eGFR). Scatterplots compare eGFR to log[(E_Cd_/C_cr_) × 10^5^] in subjects grouped by locality (**A**–**C**) and in all subjects (**D**). Quadratic and linear coefficients of determination (*R*^2^) are provided together with corresponding equations, standardized β coefficients, and *p*-values.

Figure 1A–D present scatterplots of eGFR against log(E_Cd_/C_cr_) in each exposure subset and the entire sample. In each subset, significant linear and quadratic relationships were documented, and quadratic *R*^2^ values were slightly higher. Quadratic *R*^2^ was 0.228 in the low-Cd group, 0.083 in the moderate-Cd group, 0.154 in the high-Cd group, and 0.378 in the entire sample. In the linear model, standardized β was −0.467 in the low-Cd group, −0.259 in the moderate-Cd group, −0.361 in the high-Cd group, and −0.598 in the entire sample.

**Figure 2:**
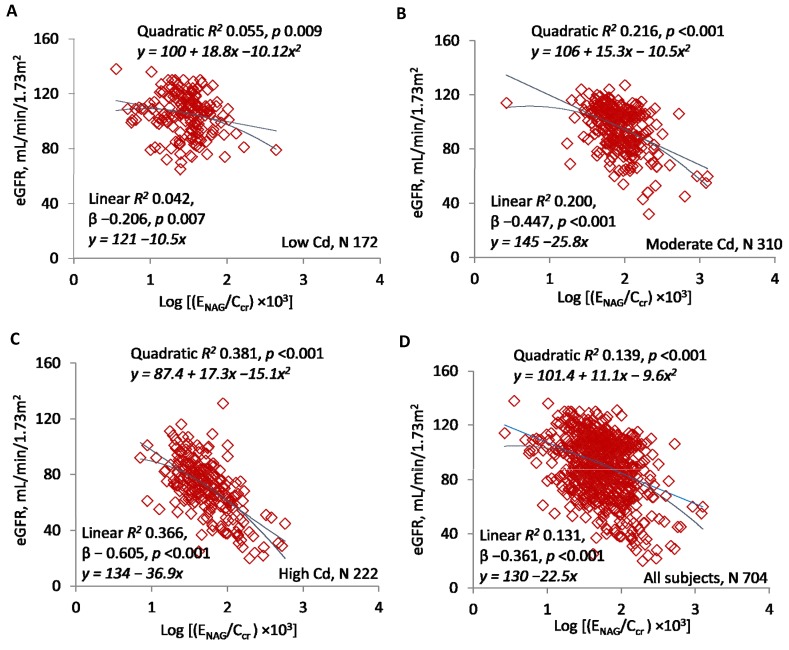
E_NAG_/C_cr_ as a predictor of eGFR. Scatterplots compare eGFR to log[(E_NAG_/C_cr_) × 10^3^] in subjects grouped by locality (**A**–**C**) and in all subjects (**D**). Quadratic and linear coefficients of determination (*R*^2^) are provided together with corresponding equations, standardized β coefficients, and *p*-values.

Figure 2A–D present scatterplots of eGFR against log(E_NAG_/C_cr_). As in Figure 1, significant, inverse linear and quadratic relationships were documented in subsets and the entire sample, and quadratic *R*^2^ values were slightly higher. Quadratic *R*^2^ was 0.055 in the low-Cd group, 0.216 in the moderate-Cd group, 0.381 in the high-Cd group, and 0.139 in the entire sample. In the linear model, standardized β was −0.206 in the low-Cd group, −0.447 in the moderate-Cd group, −0.605 in the high-Cd group, and −0.361 in the entire sample.

The quadratic curves in Figure 1D and Figure 2D indicated that slopes describing rates of GFR reduction varied over the ranges of log[(E_Cd_/C_cr_) × 10^5^] and log[(E_NAG_/C_cr_) × 10^3^]. We assumed that log[(E_Cd_/C_cr_) × 10^5^] of 3.0 and log[(E_NAG_/C_cr_) × 10^3^] of 1.5 represented the excretion rates of Cd and NAG at which the rates of GFR reduction increased. Table 2 confirms that the slopes changed significantly at these points on the *x*-axes.

**Figure 3:**
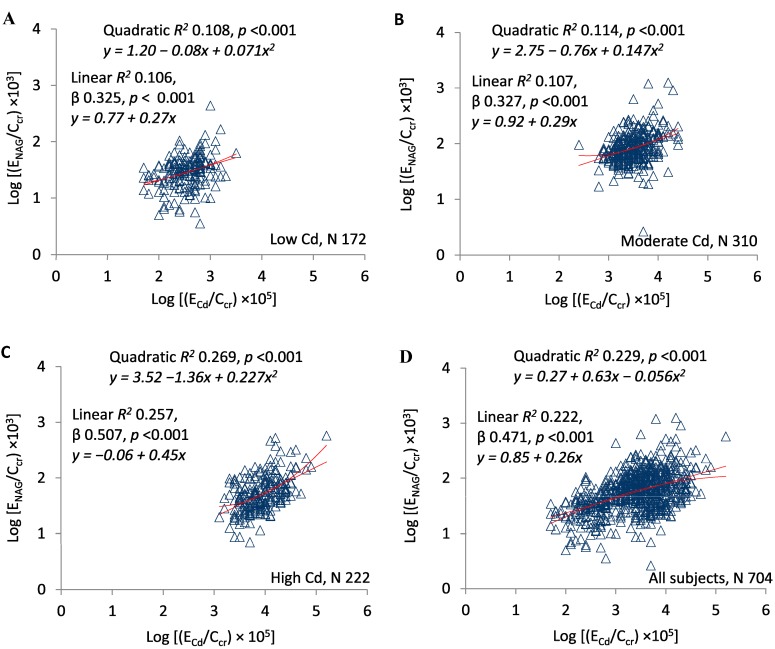
E_Cd_/C_cr_ as a predictor of E_NAG_/C_cr_. Scatterplots compare log[(E_NAG_/C_cr_) × 10^3^] to log[(E_Cd_/C_cr_) × 10^5^] in subjects grouped by locality (**A**–**C**) and in all subjects (**D**). Quadratic and linear coefficients of determination (*R*^2^) are provided together with corresponding equations, standardized β coefficients, and *p*-values.

Figure 3A–D present scatterplots of log(E_NAG_/C_cr_) against log(E_Cd_/C_cr_) in the exposure subsets and the entire sample. In all subsets, the two ratios varied directly, significant linear and quadratic relationships were documented, and quadratic *R*^2^ values were slightly higher. Quadratic *R*^2^ was 0.108 in the low-Cd group, 0.114 in the moderate-Cd group, 0.269 in the high-Cd group, and 0.229 in the entire sample. In the linear model, standardized β was 0.325 in the low-Cd group, 0.327 in the moderate-Cd group, 0.507 in the high-Cd group, and 0.471 in the entire sample.

**Figure 4:**
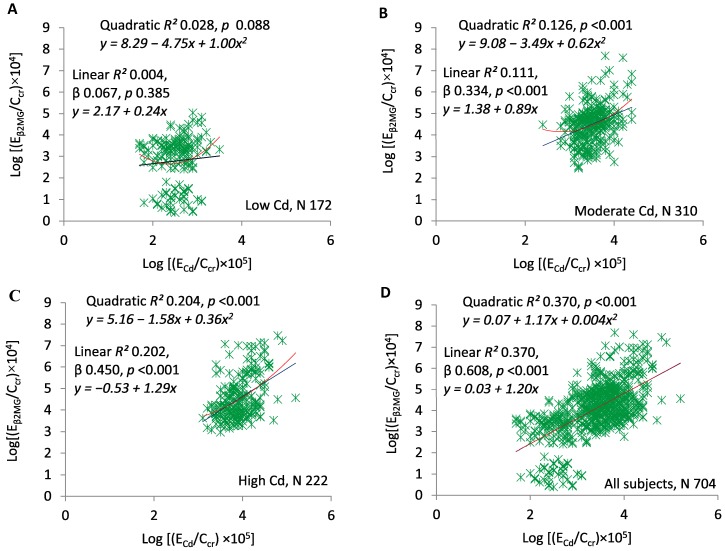
ECd/C_cr_ as a predictor of E_β2MG_/C_cr_. Scatterplots compare log[(E_β2MG_/C_cr_) × 10^4^] to log[(E_Cd_/C_cr_) × 10^5^] in subjects grouped by locality (**A**–**C**) and in all subjects (**D**). Quadratic and linear coefficients of determination (*R*^2^) are provided together with corresponding equations, standardized β coefficients, and *p*-values.

Figure 4A–D present scatterplots of log(E_β2MG_/C_cr_) against log(E_Cd_/C_cr_) in the exposure subsets and the entire sample. Figure 4A demonstrates the absence of a relationship at the lowest Cd exposure (quadratic *R*^2^ = 0.028, *p* = 0.088). At moderate and high exposure, the two ratios were directly related, significant linear and quadratic relationships were documented, and quadratic *R*^2^ values were slightly higher (Figure 4B,C). Quadratic *R*^2^ was 0.126 in the moderate exposure group, 0.204 in the high exposure group, and 0.370 in the entire sample. Quadratic and linear relationships in the entire sample were virtually identical (Figure 4D). In the linear model, standardized β was 0.067 in the low-Cd group, 0.334 in the moderate-Cd group, 0.450 in the high-Cd group, and 0.608 in the entire sample.

**Figure 5:**
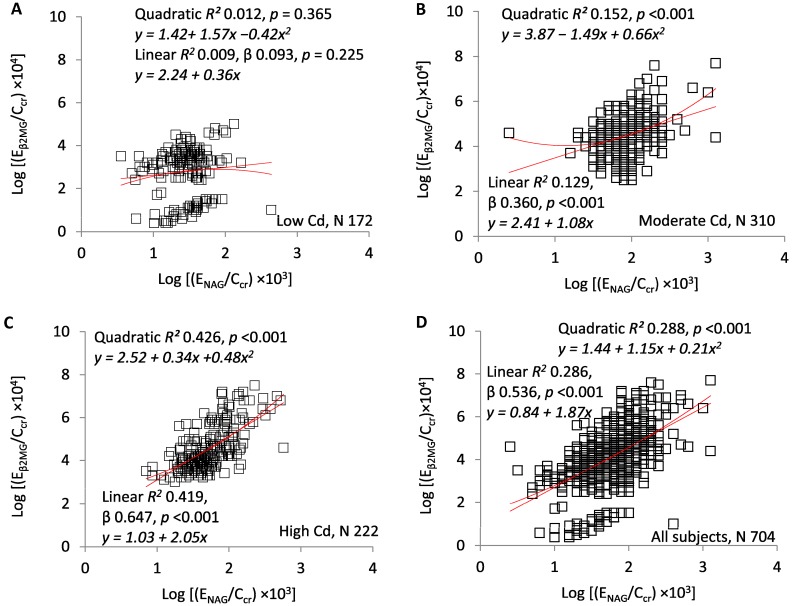
E_NAG_/C_cr_ as a predictor of E_β2MG_/C_cr._ Scatterplots compare log[(E_NAG_/C_cr_) × 10^3^] to log[(E_β2MG_/C_cr_) × 10^4^] in subjects grouped by locality (**A**–**C**) and in all subjects (**D**). Quadratic and linear coefficients of determination (*R*^2^) are provided together with corresponding equations, standardized β coefficients, and *p*-values.

Figure 5A–D present scatterplots of log(E_β2MG_/C_cr_) against log(E_NAG_/C_cr_). Figure 5A demonstrates the absence of a relationship at the lowest Cd exposure (linear *R*^2^ = 0.009, *p* = 0.225). At moderate and high exposure, the two ratios were directly related, significant linear and quadratic regressions were documented, and quadratic R^2^ values were slightly higher (Figure 5B,C). Quadratic *R*^2^ was 0.152 in the moderate exposure group, 0.426 in the high exposure group, and 0.288 in the entire sample. In the linear model, standardized β was 0.093 in the low-Cd group, 0.360 in the moderate-Cd group, 0.647 in the high-Cd group, and 0.536 in the entire sample.

## 4. Discussion

Our goals in the present study were to elucidate the source of urinary Cd and to relate that source to the pathogenesis of Cd nephropathy. Data were obtained from clinically healthy Thai subjects residing in areas with low, moderate, or high exposure to Cd. In Mae Sot District, Tak Province, intensity of exposure was determined from the Cd content of rice grains [35,36]. Exposure in Bangkok was assumed to be low on the basis of food analyses and dietary histories [33].

Subjects in the three subsets were demographically dissimilar (Table 1). Both age and percentage of smokers rose with intensity of exposure. The percentage of women was particularly high in the moderate exposure group, and some women were of childbearing age. Because iron and Cd share a transporter in intestinal epithelium, menstruating women in this group may have absorbed Cd with exceptional avidity and incurred exceptional tubular toxicity secondarily (Table 1). In the high exposure subset, increased age may have conferred additional reasons for deterioration of GFR, and smoking may have provided a second environmental source of Cd. Whether smoking itself could have accelerated the progression of CKD is unresolved [40,41,42]. Neither age, nor smoking per se, nor the source of exogenous Cd obscured the significant relationship between eGFR and E_Cd_/C_cr_.

Table 1 shows that with the increasing intensity of exposure, E_Cd_/C_cr_ rose and eGFR fell in stepwise fashion. In contrast, both E_NAG_/C_cr_ and E_β2MG_/C_cr_ were higher in the moderate than in the high exposure group. Although one might expect a direct relationship between NAG excretion and the number of intact nephrons, the higher E_NAG_/C_cr_ in the moderate group implies that the median excretion of NAG *per intact nephron* was also higher in these subjects. At the same time, the overlap of E_NAG_/C_cr_ between the moderate and high exposure groups was substantial (Figure 2 and Figure 5), and consistent relationships among E_Cd_/C_cr_, E_NAG_/C_cr_, and eGFR were demonstrable at all intensities of exposure (Figure 1, Figure 2 and Figure 3). We speculate that the number of menstruating women in the moderate exposure subset was sufficient to increase Cd absorption and tubular toxicity in the entire group, but insufficient to disrupt the statistical relationships seen in all groups among E_Cd_/C_cr_, E_NAG_/C_cr_, and eGFR.

Analogous statements can be made about β_2_MG. As impaired reabsorption of this protein is an early sign of proximal tubular injury, it is not surprising that median E_β2MG_/C_cr_ tracked with median E_NAG_/C_cr_ (Table 1). As would be expected, E_β2MG_/C_cr_ varied directly with E_NAG_/C_cr_ in the moderate and high exposure groups, but also varied directly with E_Cd_/C_cr_, which increased progressively with the intensity of exposure (Figure 4 and Figure 5).

Although CKD-EPI equations estimate GFR imprecisely [37,38], each exposure group differed significantly from the others with respect to eGFR (Table 1). Estimated GFR was inversely related to E_Cd_/C_cr_ and E_NAG_/C_cr_ in the entire sample and each subset (Figure 1 and Figure 2), and E_NAG_/C_cr_ varied directly with E_Cd_/C_cr_ (Figure 3). For all comparisons in Figure 1, Figure 2 and Figure 3, both linear and quadratic relationships were significant, and with one exception (Figure 1B), R^2^ rose with the exposure intensity. Standardized β followed the same pattern. Despite the statistical significance of all comparisons, some R^2^ values indicated that the fractional contribution of E_Cd_/C_cr_ or E_NAG_/C_cr_ to eGFR was <10% (Figure 1B and Figure 2A); simultaneously, however, standardized β indicated robust effects of changes in *x* on changes in *y*. Factors other than E_Cd_/C_cr_ and E_NAG_/C_cr_ affected eGFR, but variation in each ratio was associated with substantial variation in eGFR.

Taken together, the graphs in Figure 1, Figure 2 and Figure 3 imply that in each subset, GFR was inversely related to the severity of cellular injury per nephron (E_NAG_/C_cr_), which in turn was associated with the amount of Cd excreted per nephron (E_Cd_/C_cr_). In addition, the quadratic relationships in Figure 1 and Figure 2 suggest that small increments in the most advanced injury were accompanied by disproportionate reductions in GFR. Slope analyses of curves in Figure 1D and Figure 2D confirm this inference (Table 2).

Other investigators have described direct relationships of [NAG]_u_/[cr]_u_ and eGFR to [Cd]_u_/[cr]_u_, but we have not found a synthesis of those relationships into a satisfactory pathophysiologic narrative [35,43,44,45,46,47,48]. A cogent interpretation of Figure 1, Figure 2 and Figure 3 must explain how eGFR and E_NAG_/C_cr_—results of *cumulative* Cd sequestration—were related physiologically to E_Cd_/C_cr_, an indicator of Cd excretion *at the time of sampling*. Cd was excreted for two possible reasons; it was filtered and not reabsorbed, or it was released from tubular cells [32]. Although both processes may have occurred, we do not see how the first, excretion after filtration, could have produced a physiologic connection between E_Cd_/C_cr_ and E_NAG_/C_cr_ (Figure 3). In contrast, if Cd was released from damaged tubules, then Cd and NAG emanated from the same source, and both E_Cd_/C_cr_ and E_NAG_/C_cr_ measured cellular injury. This shared attribute of Cd and NAG explains the statistical association of the ratios.

Additional evidence for the tubular origin of excreted Cd is provided by demonstrated extrusions of MT into tubular lumens [49], documented correlations of E_Cd_ with renal tissue content of Cd [50,51,52], and direct relationships between E_Cd_ and GFR (number of intact nephrons) [53,54,55]. Experiments in rabbits demonstrated a high tubular maximum for reabsorption of CdMT that would preclude excretion of filtered Cd in the typically intoxicated human [56]. 

In addition to addressing the likely source of excreted Cd, we must also ask why declining eGFR, the result of continuous loss of intact nephrons over time, was associated in the present study with parameters of *current* cellular injury, E_Cd_/C_cr_ and E_NAG_/C_cr_. To address this paradox, we propose that eGFR, E_Cd_/C_cr_, and E_NAG_/C_cr_ were simultaneous consequences of the tubular content of Cd. As the content rose, cellular injury per nephron and the rate of nephron loss increased proportionately; at any moment in a subject’s exposure history, the three variables were quantitatively associated because they were traceable to the same burden of sequestered Cd.

β_2_MG, a small protein made by nucleated cells, is almost completely filtered by glomeruli [29]. Ordinarily, the proximal tubule reabsorbs and degrades over 99% of filtered β_2_MG [30]. Because tubulopathies increase E_β2MG_ [31], excessive E_β2MG_ is conventionally interpreted as evidence of reabsorptive dysfunction [35,57,58]. This interpretation is understandable, but we suspect that it is an oversimplification. One reason is that endogenous production of β_2_MG may be increased by chronic inflammatory conditions, solid tumors, lymphatic malignancies, and multiple myeloma [59]. If tubular degradation (TD_β2MG_) remains constant as production rises, E_β2MG_ also rises even though TD_β2MG_ has not fallen (SM). Moreover, if both β_2_MG production and TD_β2MG_ per volume of filtrate (TD_β2MG_/C_cr_) remain constant as GFR falls, TD_β2MG_ also falls, and E_β2MG_ rises (SM). Although these inferences are unproven, it seems likely that a combination of reabsorptive dysfunction and reduced GFR caused associations of E_β2MG_/C_cr_ with E_Cd_/C_cr_ and E_NAG_/C_cr_ (Figure 4 and Figure 5).

An additional observation requires explanation. In the low exposure group, a cluster of subjects exhibited exceptionally low E_β2MG_/C_cr_. At a fixed rate of β_2_MG filtration (equal to endogenous production) and a fixed value of TD_β2MG/Ccr_, the rate of β_2_MG reabsorption increases with the number of intact nephrons, and E_β2MG_ decreases simultaneously. In the isolated cluster, mean eGFR was 105.3 mL/min/1.73 m^2^; in the remainder of subjects in the study, it was 89.3 mL/min/1.73 m^2^. We suspect that extremely low E_β2MG_/C_cr_ in the cluster was the result of high C_cr_, high TD_β2MG_, and secondarily reduced E_β2MG_.

In the present study, we have continued the recently introduced practice of normalizing excretion of Cd, NAG, and β_2_MG to creatinine clearance instead of creatinine excretion [26]. Because the resulting ratios express E_Cd_, E_NAG_, and E_β2MG_ as functions of intact nephron mass, they nullify sources of imprecision that accompany normalization to E_cr_ or [cr]_u_. At any GFR, E_cr_ is primarily a function of muscle mass [60]; consequently, at a given E_Cd_ (for example), [Cd]_u_/[cr]_u_ may vary by a multiple over the range of human body size. Moreover, multiple groups have reported *direct* rather than inverse relationships between GFR and [Cd]_u_/[cr]_u_ after Cd exposure [5,53,54,55]. If the nephron number determines E_Cd_ at a given cellular burden of the metal, then [Cd]_u_/[cr]_u_ may exaggerate the burden at normal GFR and underestimate it at reduced GFR. Normalization of E_Cd_ to C_cr_—that is, calculation of [Cd]_u_[cr]_p_/[cr]_u_—eliminates the confounding effects of both muscle mass and nephron number on [Cd]_u_/[cr]_u_. In addition, because the required measurements are made in aliquots of urine and serum, the calculation quantifies amounts of Cd (or other substances) excreted per volume of filtrate while eliminating the need for timed urine collections and direct determinations of GFR. We plan to address optimal expression of excretion rates relevant to Cd nephropathy in a separate publication.

In summary, we draw the following conclusions from the significant regressions described herein. E_NAG_/C_cr_ varied directly with E_Cd_/C_cr_ because sequestered Cd induced the release of NAG and Cd from tubular cells into filtrate. Estimated GFR varied inversely with both ratios because all three parameters reflected the extent of tubular Cd accumulation. E_NAG_/C_cr_ and E_Cd_/C_cr_ quantified ongoing cellular injury, and eGFR quantified the loss of intact nephrons. We suspect that the significant regressions of E_β2MG_/C_cr_ on E_Cd_/C_cr_ and E_NAG_/C_cr_ resulted from effects of Cd on both tubular reabsorption and nephron number.

## Figures and Tables

**Table 1:** Study subjects drawn from three localities.

Descriptors	All Subjects	Locality		
		Low Cd	Moderate Cd	High Cd
Number of subjects	704	172	310	222
Women (%)	60.7	47.7	72.9	53.6 *
Smoking (%)	43.6	23.8	40.6	63.1 *
Age (years)	48.34 ± 11.11	38.72 ± 10.29	47.24 ± 4.72	57.34 ± 11.13 ^†^
SBP (mmHg)	122.7 ± 13.5	120.1 ± 10.4	124.2 ± 14.7 ¶	−
DBP (mmHg)	78.9 ± 9.5	78.1 ± 7.6	79.4 ± 10.4	−
MBP (mmHg)	93.5 ± 9.9	92.1 ± 7.9	94.3 ± 3.3 ¶¶	−
eGFR (mL/min/1.73 m^2^)	90.32 ± 21.84	105.46 ± 15.05	95.72 ± 16.13	71.05 ± 19.65 ^†^
CKD prevalence (%)	9.5	0	3.5	25.2
Kidney disease stage (%)				
Stage 1	54.8	84.1	66.5	16.2 *
Stage 2	36.6	15.9	31.0	59.9 *
Stage 3	7.7	0	2.6	20.7 **
Stage 4	1.0	0	0	3.2
Serum creatinine (mg/dL)	0.80 (0.70, 1.0)	0.8 (0.7, 0.9)	0.8 (0.7, 0.9)	1.0 (0.9, 1.2) ^†^
Urine creatinine (mg/dL)	97 (53, 156)	52 (32, 106)	110 (62, 171)	115 (69, 158) ^†^
Urine Cd (μg/L)	3.7 (1.1, 8.3)	0.2 (0.1, 0.6)	3.9 (2.4, 7.2)	8.3 (4.7, 13.9) ^†^
Urine NAG (units/L)	6.4 (2.4, 16.6)	1.8 (1.3, 2.9)	11.9 (7.1, 19)	5.3 (2.7, 9.2) ^†^
Urine β_2_MG (μg/L)	154 (33, 778)	9.5 (0.3, 42)	400 (134, 1118)	171 (64, 1368) ^†^
E_Cd_/C_cr_ × 100, µg/L	3.1 (1.1, 7.0)	0.3 (0.3, 0.6)	3.0 (1.7, 5.0)	8.2 (4.8, 15) ^†^
E_NAG_/C_cr_ × 100, units/L	5.6 (3.4, 9.4)	3.2 (2.0, 4.1)	8.0 (5.8, 12)	4.9 (3.1. 8.3) ^†^
E_β2MG_/C_cr_ × 100, µg/L	137 (29, 604)	17 (0.4, 36)	368 (92, 808)	162 (64, 176) ^†^

SBP = systolic blood pressure; DBP = diastolic blood pressure; MBP = Mean arterial pressure; CKD = chronic kidney disease; eGFR = estimated glomerular filtration rate; NAG = N-acetyl-β-d-glucosaminidase. MBP = DBP + (pulse pressure)/3, where pulse pressure = SBP − DBP. Data for age and eGFR are arithmetic mean values ± standard deviation (SD). Data for blood pressure are geometric mean values ± SD. Data for all other continuous variables are the median (25th, 75th percentile) values. * Significant % differences among three groups (*p* < 0.05, Pearson Chi-Square test). ** Significant % differences between two groups (*p* < 0.001, Pearson Chi-Square test). ^†^ Significant mean differences among three groups (*p* < 0.001, Kruskal–Wallis test). ¶ Significant difference from the low exposure group (*p* = 0.003, Mann–Whitney U-test). ¶¶ Significant difference from the low exposure group (*p* = 0.014, Mann–Whitney U-test).

**Table 2:** Slope analysis for comparing rates of eGFR reduction.

Excretion Rates of Cd or NAG	Number of Subjects	eGFR vs. log[(E_Cd_/C_cr_) × 10^5^] or log[(E_NAG_/C_cr_) × 10^3^]			
		β Coefficients		*R* ^2^	*p* Value
		Slope (Unstandardized β) ± SE	Standardized β		
Log[(E_Cd_/C_cr_) × 10^5^]					
<3	168	−17.62 ± 3.20	−0.392	0.154	<0.001
≥3	536	−27.71 ± 2.06	−0.503	0.253	<0.001
All subjects	704	−20.86 ± 1.06	−0.598	0.357	<0.001
Log[(E_NAG_/C_cr_) × 10^3^]					
<1.5	153	−15.91 ± 7.62	−0.168	0.028	0.038
≥1.5	551	−28.35 ± 3.35	−0.339	0.115	<0.001
All subjects	704	−22.49 ± 2.19	−0.361	0.131	<0.001

The standardized β coefficient indicates the strength of the association of eGFR with log[(E_Cd_/C_cr_) × 10^5^] or log[(E_NAG_/C_cr_) × 10^3^]. *R*^2^ values are coefficients of determination that indicate the fraction of eGFR variation explained by E_Cd_/C_cr_ or E_NAG_/C_cr_. *p* ≤ 0.05 identifies statistically significant eGFR reduction rates or associations of eGFR with urinary Cd or NAG excretion.

## Supplementary Materials

The following are available online at https://www.mdpi.com/2305-6304/7/4/55/s1, The equation for normalization to creatinine clearance.

## Abbreviations

**Abbreviation**	**Meaning**
Cd	Cadmium
GFR	Glomerular filtration rate, units of volume/time
eGFR	Estimated glomerular filtration rate, units of mL/min/1.73 m^2^
CKD-EPI	Chronic kidney disease epidemiology collaboration
MT	Metallothionein
CdMT	Cadmium–metallothionein complex
PC	Phytochelatin
CdPC	Cadmium–phytochelatin complex
GSH	Glutathione
NAG	N-acetyl-β-d-glucosaminidase
β_2_MG	Beta_2_-microglobulin
C_cr_	Creatinine clearance, units of volume/time
V_u_	Urine flow rate, units of volume/time
E*_x_*/C_cr_	Excretion rate of *x* per volume of filtrate, units of mass/volume, where *x* = Cd, NAG, or β_2_MG
TD_β2MG_	Rate of tubular degradation of β_2_MG, units of mass/time
TD_β2MG_/C_cr_	Amount of β_2_MG degraded per volume of filtrate, units of mass/volume
