# Supplementary material for: The Source and Pathophysiologic Significance of Excreted Cadmium
Source: Toxics. 2019 Oct 18;7(4):55. doi: 10.3390/toxics7040055 (PMC6958378; doi:10.3390/toxics7040055)
Supplement: Supplementary file 1 [file toxics-07-00055-s001.pdf]

Supplementary Material: The Source and Pathophysiologic Significance of Excreted Cadmium

Soisungwan Satarug, David A. Vesey, Werawan Ruangyuttikarn, Muneko Nishijo, Glenda C. Gobe and Kenneth R. Phelps

1. Demonstration that $E_x/C_{cr} = [x]_u[cr]_p/[cr]_u$.

Let V_u = urine flow rate, units of volume/time;
E_x = urinary excretion rate of substance *x*, units of mass/time;
$[x]_u$ = urinary concentration of substance *x*, units of mass/volume;
E_{cr} = urinary excretion rate of creatinine, units of mass/time;
$[cr]_p$ = plasma concentration of creatinine, units of mass/volume;
$[cr]_u$ = urine concentration of creatinine, units of mass/volume;
C_{cr} = renal creatinine clearance (an approximation of GFR) = $E_{cr}/[cr]_p$, units of volume/time;
E_x/C_{cr} = amount of *x* excreted per volume of filtrate, units of mass/volume.
$E_x/C_{cr} = [x]_uV_u/([cr]_uV_u/[cr]_p)$; cancelling V_u and rearranging,
$E_x/C_{cr} = [x]_u[cr]_p/[cr]_u$.

2. Demonstration that E_{Cd}/C_{cr} is Unaffected by Muscle Mass

Let
(a) V_u = urine flow rate
(b) $E_{Cd} = [Cd]_uV_u$;
(c) $E_{cr} = [cr]_uV_u$; and
(d) $C_{cr} = E_{cr}/[cr]_p = [cr]_uV_u/[cr]_p$.

E_{cr} is directly related to muscle mass [60]. According to equation (d), at a given C_{cr}, E_{cr} and $[cr]_p$ rise or fall by the same factor.

If E_{Cd} is normalized to E_{cr}, then $E_{Cd}/E_{cr} = [Cd]_uV_u/[cr]_uV_u$. Since E_{cr} is directly related to muscle mass, E_{Cd}/E_{cr} is inversely related to muscle mass at any E_{Cd}. The same is true of $[Cd]_u/[cr]_u$ after cancellation of V_u in the numerator and denominator.

If E_{Cd} is normalized to C_{cr}, then E_{Cd}/C_{cr}, *i.e.*, $[Cd]_uV_u/[cr]_uV_u/[cr]_p$, is unaffected by muscle mass because *at a given* C_{cr} = ($E_{cr}/[cr]_p$), E_{cr} and $[cr]_p$ rise or fall by the same factor as muscle mass varies. This fact remains true after simplification of the complex fraction to yield $E_{Cd}/C_{cr} = [Cd]_u[cr]_p/[cr]_u$.

3. Demonstration that $E_{\beta 2MG}$ May Rise because of Increased Endogenous Production

Let $I_{\beta 2MG}$ = influx of β_2MG from endogenous sources into plasma;
$[\beta_2MG]_p$ = plasma concentration of β_2MG, mg/L;
$F_{\beta 2MG}$ = rate of glomerular filtration of β_2MG, mg/d;
$E_{\beta 2MG}$ = urinary excretion rate of β_2MG, mg/d;
$TD_{\beta 2MG}$ = rate of tubular degradation of β_2MG, mg/d;
GFR = glomerular filtration rate, L/d.
Assume that $F_{\beta 2MG} = GFR[\beta_2MG]_p = E_{\beta 2MG} + TD_{\beta 2MG}$.

Assume an equilibrium between $I_{\beta 2MG}$ and $F_{\beta 2MG}$, and assume stable GFR. If $I_{\beta 2MG}$ rises, so do $[\beta_2MG]_p$ and $F_{\beta 2MG}$. If $TD_{\beta 2MG}$ remains stable, $E_{\beta 2MG}$ must rise if $F_{\beta 2MG}$ rises. Thus $E_{\beta 2MG}$ may rise even though $TD_{\beta 2MG}$ has not fallen.

4. Demonstration that $E_{\beta 2MG}$ Rises if GFR Falls

From item 3, $F_{\beta 2MG} = GFR[\beta_2MG]_p = E_{\beta 2MG} + TD_{\beta 2MG}$. Dividing the equation on the right by GFR, $[\beta_2MG]_p = E_{\beta 2MG}/GFR + TD_{\beta 2MG}/GFR$.

Assume that $I_{\beta 2MG}$ and thus $F_{\beta 2MG}$ remain stable. Since $F_{\beta 2MG}$ = GFR[β2MG]$_p$, [β2MG]$_p$ must rise reciprocally if GFR falls. Assume that $TD_{\beta 2MG}$/GFR remains stable as GFR falls. If GFR has fallen, $TD_{\beta 2MG}$ must also fall. Since $F_{\beta 2MG}$ is constant and $F_{\beta 2MG} = E_{\beta 2MG} + TD_{\beta 2MG}$, $E_{\beta 2MG}$ must rise.
